# Analysis of Factors Influencing Relapse and Pregnancy in Patients with Borderline Ovarian Tumors

**DOI:** 10.7150/jca.56387

**Published:** 2021-07-02

**Authors:** Ya Qi, Min Wang, Yuwei Yang, Zhi Zeng, Yingying Zhou

**Affiliations:** Department of Gynecology and Obstetrics, Shengjing Hospital Affiliated of China Medical University, Shenyang, Liaoning, China.

**Keywords:** borderline ovarian tumor, recurrence, fertility preservation, pregnancy

## Abstract

**Objective:** This retrospective study analyzed the factors affecting recurrence in patients after surgery with borderline ovarian tumors and postoperative recurrence and pregnancy after fertility-sparing surgery (FSS), to provide guidance for clinical treatment of borderline ovarian tumors and propose a therapeutic strategy for fertility protection.

**Methods:** A total of 415 patients with borderline ovarian tumors were initially operated on in the gynecology ward of Shengjing Hospital Affiliated with China Medical University from September 1, 2013, to September 1, 2019. Central pathology review and prospective follow-up were carried out. The clinical and pathological data were consulted through the medical record query system of our hospital. The recurrence and pregnancy of the patients were investigated through telephone follow-up and outpatient and inpatient medical records. The influence of clinical and pathological variables on recurrence and pregnancy were evaluated using univariate/multivariate analyses.

**Results:** In this study, 415 patients were collected, of which 21 lost follow-up, and a total of 394 eligible patients were included in the analysis. Among these patients, 25 patients relapsed with a recurrence rate of 6.3% and there were 196 patients with fertility-sparing surgery, of the 63 patients attempting to conceive, 35 were able to attain pregnancy with a pregnancy rate of 55.6%. All patients survived until the follow-up deadline. In univariate and multivariate analyses, FSS, FIGO stage, and micropapillary pattern were independent risk factors for recurrence of BOTs. FIGO stage, micropapillary pattern were independent risk factors for recurrence of BOTs with FSS. The risk of recurrence was not related to omentectomy nor postoperative chemotherapy. While omentectomy and chemotherapy had an impact on the pregnancy rate (*P* <0.05) and the pregnancy rate of patients without omentectomy or chemotherapy was higher.

**Conclusion:** Omentectomy did not affect recurrence and it is not recommended as a routine operation. Adjuvant chemotherapy does not reduce the recurrence rate. While omentectomy and chemotherapy had an impact on the pregnancy rate, and both of them should be carried out more carefully in patients with fertility requirements.

## Introduction

Borderline ovarian tumor (BOT) refers to a non-proliferative disease that does not destroy the ovarian basal layer. Its histopathology and biological behavior are between benign and malignant tumors, accounting for 10% of epithelial ovarian tumors 1,2. BOTs grow slowly and most of them have a favorable prognosis. Approximately 75% of patients at the time of diagnosis are in FIGO stage I 3 and the 5-year and 10-year survival rates of BOT women with stage I are impressive, about 95% -97% and 70% -95 %, respectively 4. Even if diagnosed as an advanced stage (stage II-III), the 5-year survival rate can still reach 65-87% 4. Borderline ovarian tumor (BOT) usually occurs in young women of childbearing age with a median age of onset of 45 years. Notably, the proportion of patients under 40 years old is 34% 5. Given the reproductive age and good prognosis, fertility preservation is an important factor in planning treatment for young patients with borderline ovarian tumors 6. The etiology of BOT is multifaceted and has not been fully delineated at present, but estrogen, obesity, smoking, drinking, and heredity have been implicated 7. Infertility may also increase the risk of BOT 8. The BRCA mutation rate of BOT is lower than that of fallopian tube/ovarian cancer in terms of molecular genetics 9.

The common histopathological types of BOT are serous borderline tumor (SBOT), mucinous borderline tumor (MBOT), and other rare types such as seromucous borderline tumor, endometrioid tumor, clear cell tumor, and Brenner tumor 10. Moreover, the histological classification of SBOT includes the classical type and micropapillary type which has been reported may be associated with disease recurrence 11.

BOT is mainly treated by radical or conservative surgery according to whether fertility is retained. For the past few years, with the implementation of the two-child policy in China, more and more attention has been paid from a radical approach to more conservative treatment-fertility sparing surgery (FSS). The main issue with FSS is whether it will affect the risk of disease recurrence and postoperative pregnancy outcomes. BOT patients have a good prognosis and most of the recurrence can be treated with surgery as well. However, there is still no consensus on the influencing factors of BOT patients' recurrence. Most studies have shown that staging, invasive implantation, and FSS may affect the recurrence of BOT 12,13, but whether the micropapillary pattern, preoperative CA125 level, lymph node dissection, and omentum resection affect the recurrence is controversial. Still, factors related to obstetric outcomes after FSS remain controversial. It has been reported that the pregnancy rate may be related to age and preoperative fertility, and the pregnancy rate is higher in young patients with preoperative reproductive history 14,15.

Investigating the related factors of BOT recurrence and obstetric outcomes after FSS is important for prognosis predicting, post-operative monitoring, and appropriate counseling for patients. There have been few studies to concern the factors of recurrence and subsequent obstetric outcomes after conservative surgery.

This study retrospectively analyzed the clinicopathological and biological data of 415 patients with borderline ovarian tumors to study the influencing factors of BOT recurrence and further explore the recurrence and pregnancy of fertility-sparing surgery, to provide references for clinical treatment of BOT.

## Materials and methods

### Research object

General data was collected from clinical data of patients who met the following criteria in the gynecology ward of Shengjing Hospital affiliated to China Medical University from September 1, 2013 to September 1, 2019.

#### Inclusion criteria

Patients with initial treatment and operation in Shengjing Hospital affiliated to China Medical University. Postoperative paraffin pathology was diagnosed as borderline ovarian tumors by a blind independent reading of two physicians above the associate professor level. Complete clinical data.

#### Exclusion criteria

Patients who received initial treatment in Shengjing Hospital affiliated to China Medical University without surgery. Complicated with gynecological malignant tumors or have a history of other malignant tumors. Complicated with severe heart, lung, liver, kidney dysfunction, etc. Incomplete clinical data.

### Research methods

#### Collection of clinicopathological and biological data of study objects

The hospital medical record inquiry system was used to analyze the clinicopathological data of patients who met the inclusion criteria, such as age, smoking history (none, yes), drinking history (none, yes), obesity (BMI <25, BMI ≥25), history of estrogen use (none, yes), genetic history (none, yes), marital status (unmarried, married, divorced), sexual history (none, yes), preoperative fertility (none, yes), menopause (no, yes), chief complaint (abdominal pain and abdominal distension, touching mass, physical examination findings, others), tumor size (< 10 cm, ≥10 cm), tumor location (unilateral, bilateral), pathology (serous, mucinous, others), micropapillary pattern (none, yes), invasive implantation (no, yes), surgical approach (laparotomy, laparoscopy), lymphadenectomy (no, yes), omentectomy (no, yes), FSS (no, yes), FIGO stage (FIGO stage I, ≥FIGO stage II, unstaged), clinical stage (clinical stage I, ≥clinical stage II), and biological information: preoperative tumor marker CA125 (normal ≤35 U/ml, rise >35 U/ml), CA199 (normal ≤ 37 U/ml, rise >37 U/ml), CEA (normal ≤5 ng/ml, rise > 5 ng/ml), HE4 (normal <140 pmol/L, rise ≥ 140 pmol/L), ROMA-Before (normal <11.4%, rise ≥11.4%) and ROMA-After (normal <29.9%, rise ≥29.9%) were summarized.

#### Judgment criteria

FSS: the uterus and at least one ovary were reserved. The surgical methods were divided into unilateral salpingo-oophorectomy (USO), unilateral cystectomy (UC), bilateral cystectomy (BC), and ipsilateral adnexectomy combined with contralateral ovarian cystectomy (USO+CC). Excision of visual lesions, multiple peritoneal biopsies, greater omentum excision or biopsies, and peritoneal flushing cytology were performed at the same time; that is, staging operations to preserve fertility function.

Surgery without preserving reproductive function: including total hysterectomy + bilateral adnexectomy. Peritoneal irrigation + resection of extra-ovarian lesions, greater omentum resection + peritoneal exploratory multi-point biopsy, and MBOT appendectomy at the same time, that is, complete staging operation.

The patients who met the inclusion criteria were staged according to their clinicopathological data and surgical records according to the surgical and pathological stages of ovarian cancer, fallopian tube cancer, and primary peritoneal cancer (FIGO, 2014) 16.

Micropapillary pattern serous borderline ovarian tumor (MP-SBOT) is a special type of SBOT. The diagnostic criteria include the fused micropapillary structure diameter of the tumor> 5 mm, and the atypical tumor cell nucleus is more obvious than ordinary SBOT. MP-SBOT is more prone to peritoneal implanted lesions, has a higher recurrence rate, and has a worse prognosis than ordinary SBOT 17.

Recurrence: clinical remission was achieved after surgical treatment, and tumor markers increased 3 months later, combined with imaging diagnosis of ovarian or pelvic mass or recurrence confirmed by pathology after reoperation.

### Follow-up

Through telephone follow-up and inquiry of outpatient and inpatient medical records, we can know the postoperative condition of the patients, not only whether they are receiving adjuvant chemotherapy, whether they relapse after operations, whether they are willing to give birth, whether they are pregnant after surgery, but also the way and outcome of pregnancy.

The deadline for follow-up is December 2019.

### Data Analysis

The statistical software SPSS23.0 was used for calculation. The measurement data that conformed to the normal distribution were expressed as mean ± standard deviation, and the comparison between groups was performed by t-test. The measurement data of non-normal distribution was represented by a median, and the rank-sum test of nonparametric test was used for group comparison. The adoption rate of counting data was expressed by chi-square test and Fisher exact probability method. The variables with statistically significant differences in univariate analysis were included in the COX regression model and Logistic regression model for multivariate analysis. The difference is statistically significant at *P* <0.05.

## Results

### Situation analysis of the research object

A total of 415 patients who met the inclusion criteria were collected, of which 21 were lost to follow-up, with a loss rate of 5.1%. A total of 394 BOT patients were included in the study. The minimum age was 12 years, the maximum was 80 years, and the median was 38.5 years. Follow-up time was 4-76 months, with a median follow-up time of 36.5 months. Up to the end of the follow-up, there were no deaths. The clinicopathological and biological data of the patients and their judgment standards are shown in **Table 1.**

### Influencing factors of recurrence in patients with BOT

#### Univariate analysis of influencing factors of recurrence in patients with BOT

A total of 25 patients relapsed, with a recurrence rate of 6.3%. The shortest interval of recurrence was 3 months and the longest was 48 months. The average time of recurrence was 24.0±14.0 months. Through univariate analysis, we found that the age, preoperative fertility, tumor site, invasive implantation, FSS, and FIGO stage all affected BOT recurrence, and the differences were statistically significant (*P* <0.05). However, smoking history, drinking history, obesity, history of estrogen use, genetic history, marital status, sexual history, preoperative fertility, menopause, chief complaint, tumor size, tumor site, preoperative tumor markers CA125, CA199, CEA, HE4, ROMA-Before, ROMA-After, pathology, micropapillary pattern, invasive implantation, surgical approach, lymphadenectomy, omentectomy, FSS, FIGO stage, postoperative chemotherapy and pregnancy before recurrence all did not affect recurrence, the results showed no statistical significance (*P* > 0.05) (**Table 2**).

The median age of the recurrence group was 28 years old, which was lower than that of the non-recurrence group 40 years old. The difference between the two groups was statistically significant (Z = -3.781, *P* = 0.000). The recurrence rate of patients with no preoperative fertility was higher than that of patients with preoperative fertility (12.5% vs 2.8%), and the difference was statistically significant (χ² =14.467, *P*=0.000). In a series of 394 patients, 12 of 296 (4.1%) relapsed with unilateral tumors and 13 of 98 (13.3%) with bilateral tumors. There was a significant difference between the two groups (χ²=10.512, *P*=0.001. Postoperative paraffin pathology showed that the recurrence rates of micropapillary pattern and non-micropapillary patterns were 19.4% and 5.2% respectively, and there was a significant difference between the two groups (χ²=7.357, *P*=0.007). Pathology showed that 5 of 24 (20.8%) patients with invasive implantation relapsed, and the recurrence rate of patients without invasive implantation was 5.4%. There was a significant difference between the two groups (χ²=6.618, gt*P*=0.010). 20 of 196 (10.2%) cases relapsed of FSS and 5 of 198 (2.5%) without preserving reproductive function, the difference between the two groups was statistically significant (χ²=9.773, *P*=0.002). The recurrence rate was 2% in FIGO stage I, 19.5% in FIGO stage II and above. The difference was statistically significant (χ²=15.110, *P*=0.000) (**Table 2**).

However, we found lymphadenectomy, omentectomy, postoperative chemotherapy, and pregnancy before recurrence all did not affect recurrence, the results showed no statistical significance (*P* > 0.05) (**Table 2**).

#### Multivariate analysis of influencing factors of recurrence in patients with BOT

Variables with statistically significant differences in univariate analysis: age, preoperative fertility, tumor site, micropapillary pattern, invasive implantation, FSS, and FIGO stage were included in the multivariate Cox regression model and Logistic regression model, and the data were processed by the virtual variable method. The results were shown in **Tables 3 & 4.**

COX multivariate analysis results: FIGO stage, FSS, and micropapillary pattern were independent risk factors affecting BOT recurrence. Among them, FIGO stage II and above increased the risk of recurrence by 18.204 times compared with stage I (HR=18.204, 95% CI=4.441~74.618). The risk of FSS was 14.931 times higher than that without fertility preservation operation (HR = 14.931, 95% CI = 4.304~51.794). The risk of recurrence was 4.289 times higher in those with micropapillary patterns than in those without micropapillary patterns (HR=4.289, 95% CI=1.642~11.205).

Logistic multivariate analysis results: FIGO stage, FSS, and micropapillary pattern were independent risk factors affecting BOT recurrence. Among them, FIGO stage II and above increased the risk of recurrence by 20.406 times compared with stage I (OR=20.406, 95% CI=3.854~108.038). The risk of FSS was 7.305 times higher than that without fertility preservation operation (OR=7.305, 95% CI=1.386~38.510). The risk of recurrence was 5.965 times higher in those with micropapillary patterns than in those without micropapillary patterns (OR=5.965, 95% CI=1.794~19.828).

### Postoperative status of BOT patients with FSS

#### Postoperative recurrence of BOT patients with FSS

The recurrence rate of 4.1% of patients with preoperative fertility was lower than that of 13.8% of patients without preoperative fertility, and the difference was statistically significant (χ²=4.715, *P*=0.030). 12 of 163 (7.4%) cases recurred of unilateral tumors and 8 of 33 (24.2%) of bilateral tumors, the difference was statistically significant (*P*=0.008). Postoperative paraffin pathology showed that the recurrence rates of micropapillary patterns and no micropapillary patterns were 30.8% and 8.7%, respectively. The difference between the two groups was statistically significant (χ²=4.248, *P*=0.039). Compared with laparoscopic surgery, the recurrence rate of laparotomy was higher (14.3% vs 4.8%). The difference was statistically significant (χ²=4.752, *P*=0.029). Regarding the comparison of USO with UC, 5 cases recurred after UC with a recurrence rate of 8.9%, which was higher than that of 6.5% after USO. There was no significant difference between the two groups (χ²=0.057, *P*=0.812). Compared with USO+CC, the recurrence rate of BC was lower (18.7% vs 29.4%). There was no significant difference between the two groups (*P*=0.688). The recurrence rate of FIGO stage I patients was 6.9%, and that of stage II and above patients was 50.0%, the difference was statistically significant (*P*=0.013). The recurrence rate in mucinous BOT patients with FSS was 12.5%, and that in mucinous BOT patients with FSS was 9.3%, the difference was not statistically significant (*P*=0.512) (**Table 5**).

Variables with statistically significant differences in univariate analysis: preoperative fertility, tumor site, micropapillary pattern, surgical approach, FIGO stage, and age were included in the multivariate Cox regression model and Logistic regression model, and the data were processed by a virtual variable method. The results were shown in **Tables 6 & 7.**

COX multivariate analysis results: FIGO stage and micropapillary pattern were independent risk factors that affect the recurrence in BOT patients with FSS. Among them, FIGO stage II and above increased the risk of recurrence by 21.286 times compared with stage I (HR=21.286, 95% CI=3.622~125.103). The risk of recurrence was 4.593 times higher in those with micropapillary patterns than in those without micropapillary patterns (HR=4.593, 95% CI=1.505~14.016).

Logistic multivariate analysis results: FIGO stage and micropapillary pattern were independent risk factors that affect the recurrence in BOT patients with FSS. Among them, FIGO stage II and above increased the risk of recurrence by 25.287 times compared with stage I (OR=25.287, 95% CI=2.846~224.697). The risk of recurrence was 6.941 times higher in those with micropapillary patterns than in those without micropapillary patterns (OR=6.941, 95% CI=1.601~30.085).

#### Postoperative pregnancy of BOT patients with FSS

There were 196 patients with fertility-sparing surgery, of the 63 patients attempting to conceive, 35 were able to attain pregnancy with a pregnancy rate of 55.6%. There were 40 pregnancies, including 30 cases of full-term delivery, 1 case of premature delivery, 8 cases of abortion, and 1 case of ectopic pregnancy. Among them, 2 patients were conceived by assisted reproduction, 1 case was successful full-term pregnancy, 1 case was spontaneous abortion, and the other patients were conceived naturally. There were 5 cases of cesarean section, and the rest of the patients delivered naturally. Newborns are in good health. The median time from the end of treatment to pregnancy was 12 months. The shortest pregnancy interval was 1 month and the longest was 48 months.

Univariate analysis of factors that may affect pregnancy in those with fertility requirements was shown in **Table 8.**

The omentectomy and chemotherapy affected pregnancy, and the difference was statistically significant (*P* <0.05). The pregnancy rates of patients without omentectomy or omentectomy were 63% and 35.3% respectively, and the difference was statistically significant (*P*=0.049). Four patients received postoperative chemotherapy, all of them were not pregnant, and the pregnancy rate in the non-chemotherapy group was 59.3%. The difference was statistically significant (*P*=0.034).

## Discussion

Alvarez et al. 18 reported that the postoperative recurrence rate of BOT patients was 12% to 58%, while the recurrence rate of surgery without retaining reproductive function was only 5%. Seong and others 14 have similar reports that the recurrence rate of BOT patients after operating without retaining fertility function is about 5%, while that of FSS is 10%-20%. Most of the patients relapse in the remaining ovarian tissue, and most of the recurrence is still borderline tumors, and even FSS can be performed again 2. In this study, the total recurrence rate of BOT patients was 6.3%, and the recurrence rate of BOT patients who underwent FSS was 10.2%, which was consistent with the literature report. Although the recurrence rate of BOT patients with FSS was significantly increased, the survival rate was not affected 19,20. A meta-analysis of 74 patients concluded that the recurrence rate of FSS was significantly higher (OR= 3.87; 95%CI=1.02-12.44; *P* = 0.02), but the survival rate did not decrease (5-year survival rate: OR = 0.85; 95% CI=0.03-23.82 and 7-year survival rate: OR = 0.80; 95%CI=0.08-8.41) 21. It showed that although FSS will increase the risk of disease recurrence, it was safe and feasible.

Through retrospective analysis, we found that age, preoperative fertility, tumor site, micropapillary pattern, invasive implantation, FSS, and FIGO stage were risk factors for BOT recurrence. It has been reported that age is a related factor for recurrence in patients with BOT, and younger patients are more likely to relapse 22. The analysis of Harris et al. 23 shows that the times of pregnancy are inversely proportional to the risk of BOT. Studies by Riman et al. 24 also show that women who have given birth more than once have a lower risk of developing BOT than women who have never given birth. The results in this article suggest that the recurrence rate of patients with preoperative fertility is lower than that of patients without preoperative fertility, suggesting that preoperative fertility also affects the recurrence of BOT. UzanC et al. 25 identified bilateral tumors as a risk factor for recurrence, and the 5-year recurrence-free survival rates for patients with unilateral and bilateral tumors were 71% and 48%, respectively (*P* = 0.05). Research by Alvarez 18 shows that the recurrence rate is higher in patients with micropapillary patterns or invasive implantation. Hamdullah et al. 11 believe that FSS and micropapillary pattern are both independent risk factors for BOT recurrence. Shih et al. 26 concluded research that the age of the patient at the time of onset, the micropapillary pattern of the tumor, the presence of invasive implantation, the residual lesion, and the level of CA125 before operating are the independent risk factors for the recurrence of BOT. However, there are also reports in the literature that preoperative CA125 levels are not related to BOT recurrence 12, and this article does not suggest the correlation between preoperative tumor markers and recurrence. Park et al. 27 believe that micropapillary pattern is not an independent risk factor for BOT recurrence. However, Nayyar et al.'s studies have shown that the most important predictors of recurrence are the FIGO stage at the time of surgery, the presence of invasive implants, and the presence of visible residual lesions 8.

Through multivariate analysis, we found that FSS, FIGO stage, and micropapillary pattern are independent risk factors affecting BOT recurrence.

FIGO stage, micropapillary pattern are independent risk factors for the recurrence of BOT patients with FSS. Therefore, attention should be paid to follow-up for patients with FSS, FIGO stage II and above, and postoperative paraffin pathology indicates micropapillary pattern.

The recurrence rate of laparotomy in FSS patients is higher than in laparoscopic surgery (14.3% vs 4.8%), *P* = 0.029, the difference is statistically significant. In laparoscopic surgery, tumor rupture is more common than laparotomy. Among them, tumors with a diameter of> 10cm are more likely to rupture. However, most studies have not found the impact of tumor rupture on recurrence rate and recurrence-free survival 28. Studies have shown that laparoscopic surgery with FSS does not increase the postoperative recurrence rate compared with laparotomy surgery 15. In this study, the univariate analysis found that the recurrence rate of laparoscopic surgery was low. This may be because the application of the bag reduces the iatrogenic spread caused by tumor rupture, or it may be due to the short follow-up time, or the bias caused by the difference in staging between the two groups.

FSS has the following surgical methods: UC, USO, BC, USO + CC. Morice et al. 29 showed that unilateral appendectomy can maintain good reproductive ability, and the tumor recurrence rate is lower than unilateral cystectomy. Therefore, studies tend to recommend unilateral appendectomy for young patients with unilateral BOT who wish to maintain fertility 29,30. For patients with bilateral BOT, Studies have shown that bilateral cystectomy and unilateral appendectomy + contralateral cystectomy have no difference in recurrence rate 31. But a study by Palomba et al. 32 shows that bilateral cystectomy not only has a higher pregnancy rate than unilateral appendectomy and contralateral cystectomy but also shortens the conception time, so it is more inclined to recommend bilateral cystectomy. There is no significant difference in the recurrence rate and pregnancy rate between different surgical methods in this article.

The recurrence rate in ser BOT patients with FSS was 12.5%, and that in mucinous BOT patients with FSS was 9.3%, the difference was not statistically significant (*P*=0.512). A meta-analysis of patients concluded that the recurrence rate for FSS in mucinous BOT was higher than in other pathological tissues 33. While studies by Uzan et al. 34 also show significant differences in recurrence rate after FSS among different pathological tissues. This phenomenon could be related to the limited sample size and follow-up time in the single-center study.

Some literature reports 2 indicate that the pregnancy rate with FSS reaches 50%, and most of them can achieve natural conception. There were 35 pregnancies in this study, with a pregnancy rate of 55.6%, of which only 2 were assisted reproduction and the remaining patients were spontaneously conceived, which is consistent with the literature report. The median time from the end of treatment to pregnancy was 12 months, with the shortest time interval of 1 month and the longest time interval of 48 months. At this stage, the best time for pregnancy after BOT is still inconclusive, some scholars believe that 1-2 years after pregnancy can avoid early pregnancy to increase the risk of recurrence 35, but the ovarian function decreases with the increase of age, which may increase the rate of infertility. Therefore, it is recommended that patients with BOT can try a pregnancy within 6 to 12 months after surgery to avoid the peak of recurrence 36. For chemotherapy patients, chemotherapy drugs should be stopped for half a year to one year before pregnancy, to avoid the risk of teratogenesis 37. The four chemotherapy patients in this article were not pregnant after surgery, and the pregnancy rate in the non-chemotherapy group was 59.3%, *P* = 0.034, the difference was statistically significant. It suggests that postoperative chemotherapy is a factor that affects the pregnancy of FSS patients with fertility requirements, and the pregnancy rate is higher in patients without chemotherapy. Some studies believe that whether early or late BOT, postoperative adjuvant chemotherapy cannot reduce the relapse rate or improve survival rate 14.

It is worthy to mention that our study did not find that lymphadenectomy and omentectomy have an effect on the recurrence of BOT patients. Whether lymphadenectomy affects recurrence has been controversial. Chen et al. 12 believe that lymphadenectomy is an independent risk factor for BOT recurrence, but there are also reports in the literature that lymphadenectomy is not related to recurrence 38. The 2019 NCCN guidelines suggest that lymphadenectomy does not affect overall survival, and omentectomy may affect prognosis 39. At present, most literature does not recommend routine lymphadenectomy. Trillsch et al. 40 found that the absence of omentectomy would increase the risk of recurrence in patients with BOT. However, there are also reports in the literature that omentectomy is not related to recurrence 41. It is reported that 8%-17% of patients with a macroscopically normal omentum had microscopic disease 42. Sofiane et al. reported that occult omental disease was found in 0.0 and 60.0% of patients with stages I, II, and III, respectively. Only 3.5% of patients with mucinous BOTs had omental disease compared to 45.1% of those with serious BOTs. For the purpose of staging omental biopsies are probably sufficient in a grossly normal appearing omentum 28. The rate of pregnancy in patients without omentectomy was higher than that in patients with omentectomy (63.0% vs 35.3%), which may be due to the small scope of surgery and the low incidence of pelvic adhesions after surgery. Therefore, omentectomy is not recommended as a routine operation. Adjuvant chemotherapy does not reduce the recurrence rate and it should not be provided for patients except invasive peritoneal implantation and residual lesions. And for patients with FSS who have strong fertility requirements, avoiding adjuvant chemotherapy and omentectomy can help increase the pregnancy rate, but pay close attention to follow-up.

The limitation of this study is that this study is a single-center retrospective study, the number of included cases is limited, the follow-up time is insufficient, and a large sample randomized controlled study is still needed to verify.

## Conclusion

The total recurrence rate of BOT patients was 6.3%. Univariate analysis found that age, preoperative fertility, tumor site, micropapillary pattern, invasive implantation, FSS, and FIGO stage are factors that affect the recurrence of BOT patients. COX and Logistic multivariate analysis found that the FIGO stage, FSS, and micropapillary pattern are independent risk factors that affect BOT recurrence. COX multivariate analysis found that the recurrence risk increased by 18.204 times, 14.931 times, and 4.289 times, respectively. Logistic multivariate analysis found that the recurrence risk increased by 20.406 times, 7.305 times, and 5.965 times, respectively.

The recurrence rate of BOT patients with FSS is 10.2%. Univariate analysis found that preoperative fertility, tumor site, micropapillary pattern, surgical approach, and FIGO stage are factors that affect the recurrence of BOT patients with FSS. COX and Logistic multivariate analysis found that the FIGO stage and micropapillary pattern are independent risk factors that affect the recurrence in BOT patients with FSS. COX multivariate analysis found that the recurrence risk increased risk by 21.286 and 4.593 times, respectively. Logistic multivariate analysis found that the recurrence risk increased risk by 25.287 and 6.941 times, respectively.

There were 63 cases of BOT with fertility requirements, and the pregnancy rate was 55.6%. Univariate analysis found that omentectomy and chemotherapy are the factors affecting pregnancy in BOT patients with fertility requirements.

## Figures and Tables

**Table 1:** Clinicopathological and biological data of BOT patients

Characteristic	People	Percentage (%)
Median age (minimum, maximum)	38.5 (12, 80)	
**Smoking history**		
none	380	96.4
yes	14	3.6
**Drinking history**		
none	387	98.2
yes	7	1.8
**Obesity**		
BMI <25	307	77.9
BMI ≥25	87	22.1
**History of estrogen use**		
none	386	98.0
yes	8	2.0
**Genetic history**		
none	392	99.5
yes	2	0.5
**Marital status**		
unmarried	77	19.5
married	308	78.2
divorced	9	2.3
**Sexual history**		
none	41	10.4
yes	353	89.6
**Preoperative fertility**		
none	144	36.5
yes	250	63.5
**Menopause**		
no	306	77.7
yes	88	22.3
**Chief complaint**		
Abdominal pain and abdominal distention	132	33.5
Touching mass	32	8.1
Physical examination findings	194	49.2
Others	36	9.2
**Tumor size**		
<10 cm	236	59.9
≥10 cm	158	40.1
**Tumor site**		
Unilateral	296	75.1
Bilateral	98	24.9
**CA125**		
normal ≤35 U/ml	163	41.4
rise >35 U/ml	231	58.6
**CA199**		
normal ≤37 U/ml	311	78.9
rise >37 U/ml	83	21.1
**CEA**		
normal ≤5 ng/ml	381	96.7
rise >5 ng/ml	13	3.3
**HE4**		
normal < 140 pmol/L	375	95.2
rise ≥ 140 pmol/L	19	4.8
**ROMA-Before**		
normal <11.4%	270	88.2
rise ≥11.4%	36	11.8
**ROMA-After**		
normal <29.9%	79	89.8
rise ≥29.9%	9	10.2
**Pathology**		
Serous	228	57.9
Mucinous	113	28.7
Others	53	13.4
**Micropapillary pattern**		
none	363	92.1
yes	31	7.9
**Invasive implantation**		
no	370	93.9
yes	24	6.1
**Surgical approach**		
Laparotomy	285	72.3
Laparoscopy	109	27.7
**Lymphadenectomy**		
no	243	61.7
yes	151	38.3
**Omentectomy**		
no	203	51.5
yes	191	48.5
**FSS**		
no	198	50.3
yes	196	49.7
**FIGO stage**		
FIGO stage I	150	78.5
≥FIGO stage II	41	21.5
**Chemotherapy**		
no	351	89.1
yes	43	10.9

**Table 2:** Univariate analysis of factors affecting recurrence in patients with BOT

	No recurrence (%)	Recurrence (%)	χ^2^/Z	*P*
Median age	40	28	-3.718	0.000*
**Smoking history**				
none	357 (93.9)	23 (6.1)		0.221
yes	12 (85.7)	2 (14.3)		
**Drinking history**				
none	363 (93.8)	24 (6.2)		0.370
yes	6 (85.7)	1 (14.3)		
**Obesity**				
BMI <25	285 (92.8)	22 (7.2)	1.577	0.209
BMI ≥25	84 (96.6)	3 (3.4)		
**History of estrogen use**				
none	362 (93.8)	24 (6.2)		0.411
yes	7 (87.5)	1 (12.5)		
**Genetic history**				
none	367 (93.6)	25 (6.4)		1.000
yes	2 (100.0)	0 (0.0)		
**Marital status**				
unmarried	68 (88.3)	9 (11.7)		0.125
married	292 (94.8)	16 (5.2)		
divorced	9 (100.0)	0 (0.0)		
**Sexual history**				
none	36 (87.8)	5 (12.2)	1.651	0.199
yes	333 (94.3)	20 (5.7)		
**Preoperative fertility**				
none	126 (87.5)	18 (12.5)	14.467	0.000*
yes	243 (97.2)	7 (2.8)		
**Menopause**				
no	283 (92.5)	23 (7.5)	3.162	0.075
yes	86 (97.7)	2 (2.3)		
**Chief complaint**				
Abdominal pain and abdominal distention	120 (90.9)	12 (9.1)		0.484
Touching mass	30 (93.8)	2 (6.2)		
Physical examination findings	184 (94.8)	10 (5.2)		
Others	35 (97.2)	1 (2.8)		
**Tumor size**				
<10 cm	222 (94.1)	14 (5.9)	0.169	0.681
≥10 cm	147 (93.0)	11 (7.0)		
**Tumor site**				
Unilateral	284 (95.9)	12 (4.1)	10.512	0.001*
Bilateral	85 (86.7)	13 (13.3)		
**CA125**				
normal ≤35 U/ml	155 (95.1)	8 (4.9)	0.966	0.326
rise >35 U/ml	214 (92.6)	17 (7.4)		
**CA199**				
normal ≤37 U/ml	292 (93.9)	19 (6.1)	0.138	0.710
rise >37 U/ml	77 (92.8)	6 (7.2)		
**CEA**				
normal ≤5 ng/ml	357 (93.7)	24 (6.3)		0.579
rise >5 ng/ml	12 (92.3)	1 (7.7)		
**HE4**				
normal < 140 pmol/L	350 (93.3)	25 (6.7)	0.463	0.496
rise ≥ 140 pmol/L	19 (100.0)	0 (0.0)		
**ROMA-Before**				
normal <11.4%	249 (92.2)	21 (7.8)	0.019	0.890
rise ≥11.4%	34 (94.4)	2 (5.6)		
**ROMA-After**				
normal <29.9%	78 (98.7)	1 (1.3)		0.195
rise ≥29.9%	8 (88.9)	1 (11.1)		
**Pathology**				
Serous	211 (92.5)	17 (7.5)		0.371
Mucinous	106 (93.8)	7 (6.2)		
Others	52 (98.1)	1 (1.9)		
**Micropapillary pattern**				
none	344 (94.8)	19 (5.2)	7.354	0.007*
yes	25 (80.6)	6 (19.4)		
**Invasive implantation**				
no	350 (94.6)	20 (5.4)	6.618	0.010*
yes	19 (79.2)	5 (20.8)		
**Surgical approach**				
Laparotomy	264 (92.6)	21 (7.4)	1.815	0.178
Laparoscopy	105 (96.3)	4 (3.7)		
**Lymphadenectomy**				
no	227 (93.4)	16 (6.6)	0.061	0.805
yes	142 (94.0)	9 (6.0)		
**Omentectomy**				
no	189 (93.1)	14 (6.9)	0.214	0.643
yes	180 (94.2)	11 (5.8)		
**FSS**				
no	193 (97.5)	5 (2.5)	9.773	0.002*
yes	176 (89.8)	20 (10.2)		
**FIGO stage**				
FIGO stage I	147 (98.0)	3 (2.0)	15.110	0.000*
≥FIGO stage II	33 (80.5)	8 (19.5)		
**Postoperative chemotherapy**				
no	329 (93.7)	22 (6.3)	0.000	1.000
yes	40 (93.0)	3 (7.0)		
**Pregnancy before recurrence**				
no	337 (93.6)	23 (6.4)	0.000	1.000
yes	32 (94.1)	2 (5.9)		

Note: *Indicates that the difference is statistically significant, P <0.05.

**Table 3:** COX Multivariate analysis of factors affecting recurrence in patients with BOT

	B	S.E.	Wald	*P*	HR	95% CI	
						Lower	Upper
FIGO stage	2.902	0.720	16.252	0.000	18.204	4.441	74.618
FSS	2.703	0.635	18.147	0.000	14.931	4.304	51.794
MP	1.456	0.490	8.831	0.003	4.289	1.642	11.205

**Table 4:** Logistic multivariate analysis of factors affecting recurrence in patients with BOT

	B	S.E.	Wald	*P*	HR	95% CI	
						Lower	Upper
FIGO stage	3.016	0.850	12.578	0.000	20.406	3.854	108.038
FSS	1.989	0.848	5.497	0.019	7.305	1.386	38.510
MP	1.786	0.613	8.491	0.004	5.965	1.794	19.828

**Table 5:** Univariate analysis of factors affecting recurrence in BOT patients with FSS

	No recurrence (%)	Recurrence (%)	χ^2^/Z	*P*
Median age	30	26.5	-1.647	0.100
**Smoking history**				
none	173 (90.1)	19 (9.9)		0.352
yes	3 (75.0)	1 (25.0)		
**Drinking history**				
none	173 (89.6)	20 (10.4)		1.000
yes	3 (100.0)	0 (0.0)		
**Obesity**				
BMI <25	144 (89.4)	17 (10.6)	0.002	0.965
BMI ≥25	2 (66.7)	1 (33.3)		
**History of estrogen use**				
none	174 (90.2)	19 (9.8)		0.277
yes	7 (87.5)	1 (12.5)		
**Genetic history**				
none	175 (93.6)	20 (6.4)		1.000
yes	1 (100.0)	0 (0.0)		
**Marital status**				
unmarried	65 (87.8)	9 (12.2)		0.759
married	107 (90.7)	11 (9.3)		
divorced	4 (100.0)	0 (0.0)		
**Sexual history**				
none	35 (87.5)	5 (12.5)	0.060	0.806
yes	141 (90.4)	15 (9.6)		
**Preoperative fertility**				
none	106 (86.2)	17 (13.8)	4.715	0.030*
yes	70 (95.9)	3 (4.1)		
**Menopause**				
no	172 (90.1)	19 (9.9)		0.420
yes	4 (80.0)	1 (20.0)		
**Chief complaint**				
Abdominal pain and abdominal distention	54 (87.1)	8 (12.9)		0.802
Touching mass	14 (87.5)	2 (12.5)		
Physical examination findings	96 (91.4)	9 (8.6)		
Others	12 (92.3)	1 (7.7)		
**Tumor size**				
<10 cm	105 (89.7)	12 (10.3)	0.001	0.977
≥10 cm	71 (89.9)	8 (10.1)		
**Tumor site**				
Unilateral	151 (92.6)	12 (7.4)	6.792	0.009*
Bilateral	25 (75.8)	8 (24.2)		
**CA125**				
normal ≤35 U/ml	79 (90.8)	8 (9.2)	0.174	0.677
rise >35 U/ml	97 (89.0)	12 (11.0)		
**CA199**				
normal ≤37 U/ml	139 (90.3)	15 (9.7)	0.015	0.902
rise >37 U/ml	37 (88.1)	5 (11.9)		
**CEA**				
normal ≤5 ng/ml	171 (90.0)	19 (10.0)		0.480
rise >5 ng/ml	5 (83.3)	1 (16.7)		
**HE4**				
normal <140 pmol/L	167 (89.3)	20 (10.7)		0.602
rise ≥140 pmol/L	9 (100.0)	0 (0.0)		
**ROMA-Before**				
normal <11.4%	152 (89.4)	18 (10.6)	0.207	0.649
rise ≥11.4%	20 (95.2)	1 (4.8)		
**Pathology**				
Serous	84 (87.5)	12 (12.5)		0.512
Mucinous	68 (90.7)	7 (9.3)		
Others	24 (96.0)	1 (4.0)		
**Micropapillary pattern**				
none	167 (91.3)	16 (8.7)	4.248	0.039*
yes	9 (69.2)	4 (30.8)		
**Invasive implantation**				
no	174 (90.6)	18 (9.4)		0.053
yes	2 (50.0)	2 (50.0)		
**Surgical approach**				
Laparotomy	96 (85.7)	16 (14.3)	4.752	0.029*
Laparoscopy	80 (95.2)	4 (4.8)		
**Lymphadenectomy**				
no	154 (90.6)	16 (9.4)	0.347	0.556
yes	22 (84.6)	4 (15.4)		
**Omentectomy**				
no	145 (91.2)	14 (8.8)	1.081	0.298
yes	31 (83.8)	6 (16.2)		
**Surgical methods**				
Unilateral UC	51 (91.1)	5 (8.9)	0.057	0.812
USO	100 (93.5)	7 (6.5)		
Bilateral BC	13 (81.3)	3 (18.7)		0.688
UC+CC	12 (70.6)	5 (29.4)		
**FIGO stage**				
FIGO stage I	27 (93.1)	2 (6.9)		0.013*
≥FIGO stage II	4 (50.0)	4 (50.0)		
**Postoperative chemotherapy**				
no	169 (89.4)	20 (10.6)		1.000
yes	7 (100.0)	0 (0.0)		
**Pregnancy before recurrence**				
no	144 (88.9)	18 (11.1)	0.365	0.546
yes	32 (94.1)	2 (5.9)		

Note: *Indicates that the difference is statistically significant, *P* <0.05.

**Table 6:** COX Multivariate analysis of factors affecting recurrence in BOT patients with FSS

	B	S.E.	Wald	*P*	HR	95% CI	
						Lower	Upper
FIGO stage	3.058	0.904	11.453	0.001	21.286	3.622	125.103
MP	1.525	0.569	7.174	0.007	4.593	1.505	14.016

**Table 7:** Logistic multivariate analysis of factors affecting recurrence in BOT patients with FSS

	B	S.E.	Wald	*P*	HR	95% CI	
						Lower	Upper
FIGO stage	3.230	1.115	8.400	0.004	25.287	2.846	224.697
MP	1.937	0.748	6.704	0.010	6.941	1.601	30.085

**Table 8:** Univariate analysis of pregnancy factors in BOT patients with FSS

	Non-pregnancy (%)	Pregnancy (%)	χ^2^/Z	*P*
Median age	28.5	26	-1.291	0.197
**Smoking history**				
none	27 (43.5)	35 (56.5)		0.444
yes	1 (100.0)	0 (0.0)		
**Drinking history**				
none	28 (45.2)	34 (54.8)		1.000
yes	0 (0.0)	1 (100.0)		
**Obesity**				
BMI <25	23 (43.4)	30 (56.6)	0.001	0.969
BMI ≥25	5 (50.0)	5 (50.0)		
**History of estrogen use**				
none	27 (43.5)	35 (1.2)	0.444	
yes	1 (100.0)	0 (0.0)		
**Genetic history**				
none	28 (45.2)	34 (54.8)		1.000
yes	0 (0.0)	1 (100.0)		
**Preoperative fertility**				
none	28 (46.7)	32 (53.3)		0.247
yes	0 (0.0)	3 (100.0)		
**Chief complaint**				
Abdominal pain and abdominal distention	7 (36.8)	12 (63.2)		0.357
Touching mass	2 (100.0)	0 (0.0)		
Physical examination findings	18 (47.4)	20 (52.6)		
Others	1 (25.0)	3 (75.0)		
**Tumor size**				
<10 cm	20 (45.5)	24 (54.5)	0.060	0.806
≥10 cm	8 (42.1)	11 (57.9)		
**Tumor site**				
unilateral	19 (40.4)	28 (59.6)	1.211	0.271
bilateral	9 (56.3)	7 (43.7)		
**CA125**				
normal ≤35 U/ml	8 (38.1)	13 (61.9)	0.514	0.473
rise >35 U/ml	20 (47.6)	22 (52.4)		
**CA199**				
normal ≤37 U/ml	22 (42.3)	30 (57.7)	0.167	0.683
rise >37 U/ml	6 (54.5)	5 (45.5)		
**CEA**				
normal ≤5 ng/ml	27 (43.5)	35 (56.5)		0.444
rise >5 ng/ml	1 (100.0)	0 (0.0)		
**HE4**				
normal <140 pmol/L	26 (42.6)	35 (57.4)		0.194
rise ≥140 pmol/L	2 (100.0)	0 (0.0)		
**ROMA-Before**				
normal <11.4%	24 (42.1)	33 (57.9)		0.393
rise ≥11.4%	4 (66.7)	2 (33.3)		
**Pathology**				
serous	18 (42.9)	24 (57.1)		0.143
mucinous	4 (30.8)	9 (69.2)		
others	6 (75.0)	2 (25.0)		
**Micropapillary pattern**				
none	23 (41.8)	32 (58.2)		0.449
yes	5 (62.5)	3 (37.5)		
**Invasive implantation**				
no	27 (43.5)	35 (56.5)		0.444
yes	1 (100.0)	0 (0.0)		
**Surgical approach**				
laparotomy	13 (39.4)	20 (60.6)	0.716	0.397
laparoscopy	15 (50.0)	15 (50.0)		
**Lymphadenectomy**				
no	20 (40.0)	30 (60.0)	1.938	0.164
yes	8 (61.5)	5 (38.5)		
**Omentectomy**				
no	17 (37.0)	29 (63.0)	3.871	0.049*
yes	11 (64.7)	6 (35.3)		
**Surgical methods**				
unilateral UC	5 (33.3)	10 (66.7)	0.460	0.498
USO	14 (43.8)	18 (56.2)		
bilateral BC	6 (60.0)	4 (40.0)		1.000
UC+CC	3 (50.0)	3 (50.0)		
**FIGO stage**				
FIGO stage I	6 (50.0)	6 (50.0)		0.102
≥FIGO stage II	5 (100.0)	0 (0.0)		
**Postoperative chemotherapy**				
no	24 (40.7)	35 (59.3)		0.034*
yes	4 (100.0)	0 (0.0)		
**Recurrence before pregnancy**				
no	23 (40.4)	34 (59.6)		0.080
yes	5 (83.3)	1 (16.7)		

Note: *Indicates that the difference is statistically significant, P <0.05.
